# Supplementary material for: Stabilization of V1 interneuron-motor neuron connectivity ameliorates motor phenotype in a mouse model of ALS
Source: Nat Commun. 2024 Jun 7;15:4867. doi: 10.1038/s41467-024-48925-7 (PMC11161600; doi:10.1038/s41467-024-48925-7)
Supplement: Supplementary file 1 — Supplementary Information [file 41467_2024_48925_MOESM1_ESM.pdf]

Supplementary Information

Stabilization of V1 interneuron-motor neuron connectivity ameliorates motor phenotype in a mouse model of ALS

Santiago Mora[1], Anna Stuckert[1-2], Rasmus von Huth Friis[1*], Kimberly Pietersz[3], Gith Noes-Holt[1], Roser Montañana-Rosell[1], Haoyu Wang[2], Andreas Toft Sørensen[1], Raghavendra Selvan[4], Joost Verhaagen[3], Ilary Allodi[1-2+]*

Including:

Supplementary Figure 1-4
Supplementary Table 1

Supplementary figures:

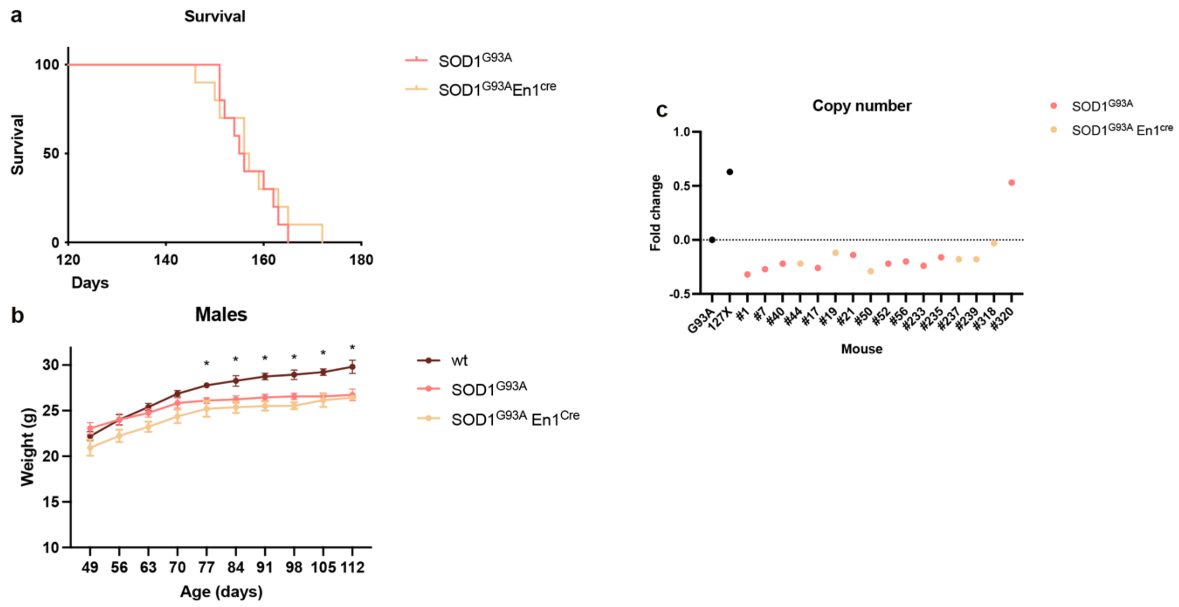

Supplementary figure 1. a) Kaplan Meyer survival curve comparing SOD1^{G93A};En1^{cre} to SOD1^{G93A} strain survival upon crossing. No significant differences were observed between the two strains (Log-rank (Mantel-Cox) test, P=0.5926, df=1, Chi square=0.2863; SOD1^{G93A};En1^{cre} N=10 mice, SOD1^{G93A} N=10 mice). Thus, disease progression is comparable between the two strains included in the study. b) Comparison of loss of weight of SOD1^{G93A}, SOD1^{G93A};En1^{cre} and WT male mice in the strains included in the study. After crossing, SOD1^{G93A};En1^{cre} do not differ from SOD1^{G93A} mice but both strains decrease in weight compared to WT littermates (two-way ANOVA and Dunnett's post hoc, F(18,110)=1.704, P=0.0489, P77 SOD1^{G93A} P=0.0307, SOD1^{G93A};En1^{cre} P=0.0046, WT N=5 mice, SOD1^{G93A} N=6 mice, SOD1^{G93A};En1^{cre} N=3 mice). Thus, weight loss is comparable in both SOD1^{G93A} and SOD1^{G93A};En1^{cre} mice. c) Copy number of human SOD1 mutations carried by experimental mice. SOD1^{G93A} fold change in pink, SOD1^{G93A};En1^{cre} in beige. First black dot depicts positive control - SOD1^{G93A} founder carrying 25 copies of the mutated gene, second black dot depicts negative control – SOD1^{127X} carrying 19 copies of the mutated gene. All graphs show mean values ± SEM.

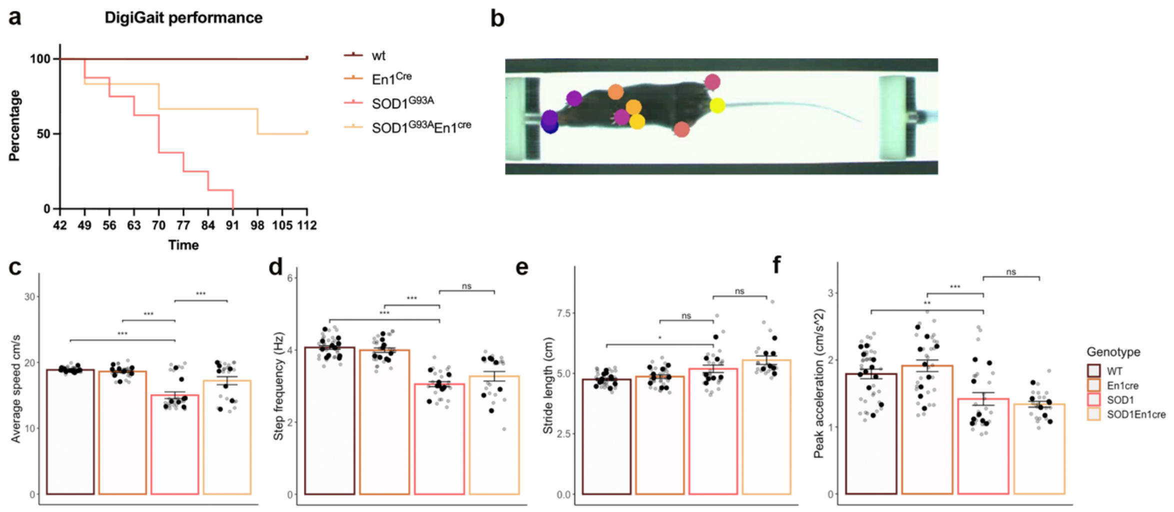

Supplementary figure 2. a) Performance of all mice included in the study on a treadmill at a speed of 20 cm/s. Percentage shows that between P49 and P63 37.5% of SOD1^{G93A} mice fail at the task, while only the 16.7% of the SOD1^{G93A};En1^{cre} cannot cope with the speed (SOD1^{G93A} median=70, SOD1^{G93A};En1^{cre} median=105, Log-rank (Mantel-Cox) test df=3, P<0.0001, Chi square=29.02, WT N=11 and SOD1^{G93A} N=8 mice, En1cre N=9, SOD1^{G93A};En1^{cre} N=7). b) Example of ventral view tracking of mice on the treadmill, 11 markers were used. c) Differences in average speed (One-way ANOVA and Dunnett's post hoc, P = 2e-04) at onset of locomotor phenotype showing that SOD1^{G93A};En1^{cre} have a later onset when compared to SOD1^{G93A} mice (WT N=11 and SOD1^{G93A} N=8 mice, En1cre N=9, SOD1^{G93A};En1^{cre} N=7). d) Step frequency, stride length e) and peak acceleration f) remain unchanged at this timepoint. All graphs show mean values ± SEM, averages values in c-f are shown in black, technical triplicates are shown in gray.

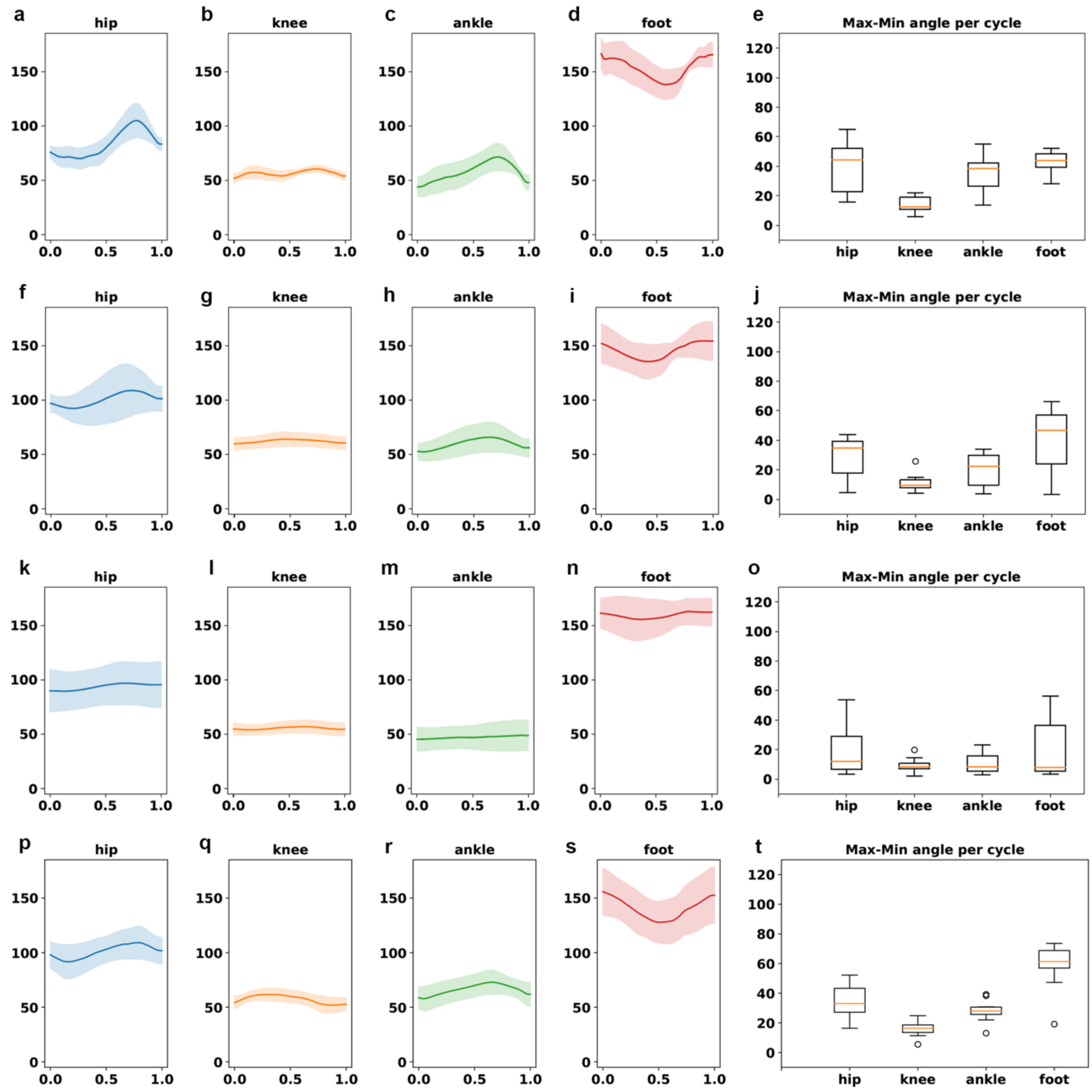

Supplementary figure 3. Example of changes in angle amplitudes within one step cycle in WT (a-e), En1^{cre} (f-j), SOD1^{G93A} (k-o) and SOD1^{G93A};En1^{cre} (p-t) mice. 15 independent steps within each video are utilized for visualization of Max-Min variability (e; j; o; t). Hip k), knee l), ankle m) and foot n) angles are reduced in the SOD1^{G93A} mice at P112 timepoint, indicative of hyperflexion which is exacerbated by disease progression. Changes in amplitude are observed in SOD1^{G93A};En1^{cre} mice overexpressing hEsyt1 (p-t).

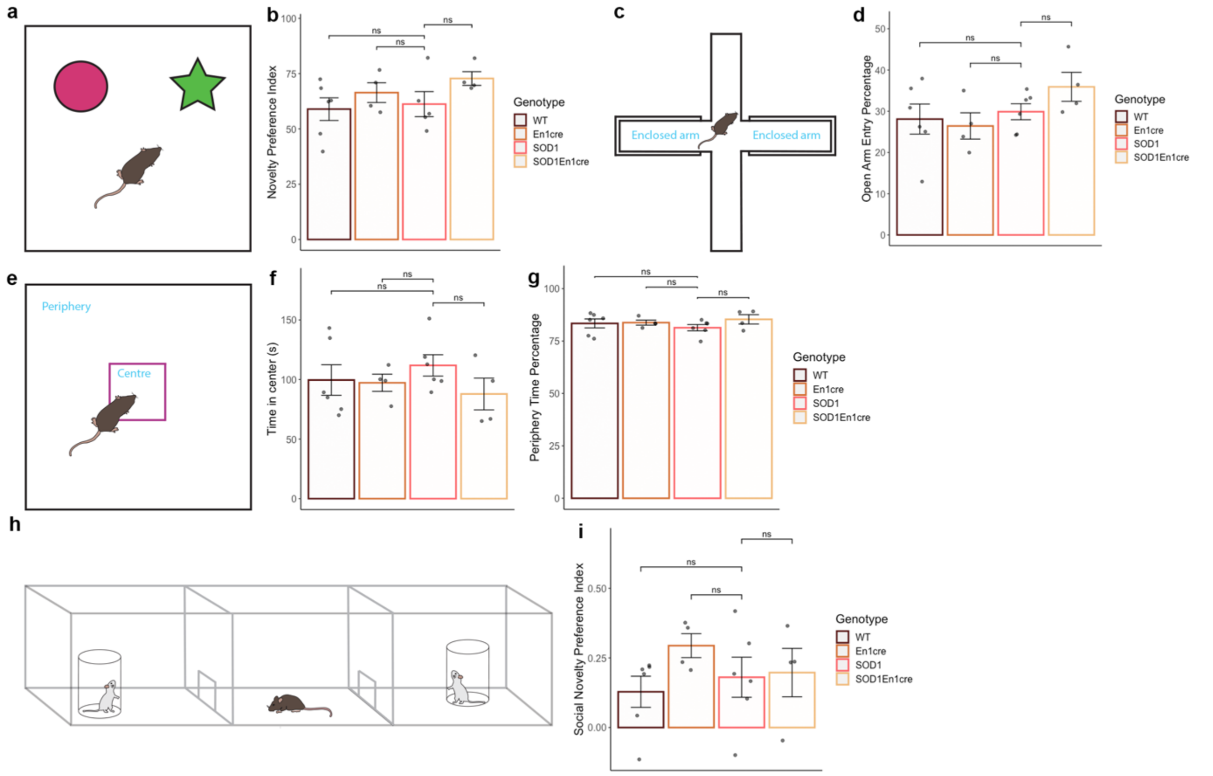

Supplementary figure 4. Cognitive assessment of WT, En1^{cre}, SOD1^{G93A} and SOD ^{G93A}En1^{cre} mice after systemic administration of viral vectors. General activity and exploration, memory, anxiety, and social behavior were investigated. a) Schematic of the novel object recognition (NOR) paradigm. b) NOR revealed no significant differences in the time spent interacting with the novel object out of the total time interacting with the objects (Novelty Preference Index) (One way ANOVA and Dunnett's post hoc: WT P = 0.9736, En1^{cre} P = 0.8236 SOD1^{G93A};En1^{cre} P = 0.3020, N = 4-6). c) Schematic of the elevated plus maze paradigm (EPM). d) SOD1^{G93A}En1^{cre} mice overexpressing hEsyt1 exhibit a higher interest in the open arms of the EPM, however no significant difference is observed for the number of entries in open arms over total arm entries (One way ANOVA and Dunnett's post hoc: WT P = 0.9490, En1^{cre} P = 0.7980, SOD1^{G93A};En1^{cre} P = 0.4357, N = 4-6). e) Schematic depicting the open field test (OFT), the purple square divides the central area from the periphery. f-g) OFT revealed no significant differences in f) time spent in center (One way ANOVA and Dunnett's post hoc: WT P =0.7487, En1^{cre} P = 0.7147, SOD1^{G93A};En1^{cre} P = 0.3574, N = 4-6) and g) time spent in the periphery out of total time (Periphery Preference) (One way ANOVA and Dunnett's post hoc: WT P = 0.7487, En1^{cre} P = 0.7153, SOD1^{G93A};En1^{cre} P = 0.3579, N = 4-6). h) Cartoon depicting experimental paradigm for the three-chamber test (3CT). No significant differences were observed in i) preference for social novelty over familiar social interaction (Social Novelty Preference Index) (One way ANOVA and Dunnett's post hoc: WT P = 0.8857, En1^{cre} P = 0.5389, SOD1^{G93A};En1^{cre} P = 0.9967, N = 4-6). Graphs show mean values ± SEM. One-way ANOVA followed by Dunnet's post-hoc test with SOD1^{G93A} as control group, N = 4-6 as indicated by individual values.

Supplementary table 1. Parameters of QuPath software for RNAscope analysis.

1.1. Endogenous Esyt1

ANALYZE > CELL DETECTION

**Setup parameters**		
Detection channel	*DAPI-T2*	
Requested pixel size	0.5 µm	
**Nucleus parameters**		
Background radius	10 um	
Median filter radius	1 µm	
Sigma	2 µm	
Minimum area	10 µm^2	
Maximum area	600 µm^2	
**Intensity parameters**		
Threshold	1	Start with 1, change as needed
Split by shape	YES	
**Cell parameters**		
Cell expansion	10 µm	
Include cell nucleus	NO	
**General parameters**		
Smooth boundaries	YES	
Make measurements	YES	

ANALYZE > CELL DETECTION > POSITIVE CELL DETECTION

**Intensity threshold parameters**		
Score compartment	*Cell: Cy3-T1 max*	
Threshold 1+	5	Start with 5, change as needed
Threshold 2+	-	
Threshold 3+	-	
Single threshold	YES	

1.2. Viral Esyt1 overexpression

ANALYZE > CELL DETECTION

**Setup parameters**	
Detection channel	*NeuroTrace -T2*
Requested pixel size	0.5 µm
**Nucleus parameters**	
Background radius	10 um

Median filter radius	1 µm	
Sigma	5-6 µm	Change as needed
Minimum area	25 μm^2	
Maximum area	800 μm^2	
**Intensity parameters**		
Threshold	200-400	Start with 200, change as needed
Split by shape	YES	
**Cell parameters**		
Cell expansion	2 µm	
Include cell nucleus	NO	
**General parameters**		
Smooth boundaries	YES	
Make measurements	YES	

113
