# Supplementary material for: Stabilization of V1 interneuron-motor neuron connectivity ameliorates motor phenotype in a mouse model of ALS
Source: Nat Commun. 2024 Jun 7;15:4867. doi: 10.1038/s41467-024-48925-7 (PMC11161600; doi:10.1038/s41467-024-48925-7)
Supplement: Supplementary file 3 — Description of Additional Supplementary Files [file 41467_2024_48925_MOESM3_ESM.pdf]

Description of Additional Supplementary Files

File Name: Supplementary Movie 1
Description: Kinematic analysis of a $SOD1^{G93A}$ mouse at P112 at a speed of 10 cm/s (average phenotype of $SOD1^{G93A}$ mice at this timepoint). Video was recorded at 150 frames/s, and it is shown at 30 frames/s.

File Name: Supplementary Movie 2
Description: Kinematic analysis of a $SOD1^{G93A}$;$En1^{cre}$ mouse at P112 at a speed of 15 cm/s (average phenotype of $SOD1^{G93A}$;$En1^{cre}$ mice at this timepoint), showing differences in hindlimb hyperflexion and in the angle of the mouse body from the belt. Video was recorded at 150 frames/s, and it is shown at 30 frames/s.
