# Supplementary material for: Stabilization of V1 interneuron-motor neuron connectivity ameliorates motor phenotype in a mouse model of ALS
Source: Nat Commun. 2024 Jun 7;15:4867. doi: 10.1038/s41467-024-48925-7 (PMC11161600; doi:10.1038/s41467-024-48925-7)
Supplement: Supplementary file 6 — Reporting Summary [file 41467_2024_48925_MOESM6_ESM.pdf]

nature portfolio

Corresponding author(s): Ilary Allodi

Last updated by author(s): Apr 24, 2024

Reporting Summary

Nature Portfolio wishes to improve the reproducibility of the work that we publish. This form provides structure for consistency and transparency in reporting. For further information on Nature Portfolio policies, see our Editorial Policies and the Editorial Policy Checklist.

Statistics

For all statistical analyses, confirm that the following items are present in the figure legend, table legend, main text, or Methods section.

n/a	Confirmed	
☐	☒	The exact sample size (*n*) for each experimental group/condition, given as a discrete number and unit of measurement
☐	☒	A statement on whether measurements were taken from distinct samples or whether the same sample was measured repeatedly
☐	☒	The statistical test(s) used AND whether they are one- or two-sided<br>*Only common tests should be described solely by name; describe more complex techniques in the Methods section.*
☒	☐	A description of all covariates tested
☐	☒	A description of any assumptions or corrections, such as tests of normality and adjustment for multiple comparisons
☐	☒	A full description of the statistical parameters including central tendency (e.g. means) or other basic estimates (e.g. regression coefficient) AND variation (e.g. standard deviation) or associated estimates of uncertainty (e.g. confidence intervals)
☐	☒	For null hypothesis testing, the test statistic (e.g. F, t, r) with confidence intervals, effect sizes, degrees of freedom and P value noted<br>*Give P values as exact values whenever suitable.*
☒	☐	For Bayesian analysis, information on the choice of priors and Markov chain Monte Carlo settings
☒	☐	For hierarchical and complex designs, identification of the appropriate level for tests and full reporting of outcomes
☐	☒	Estimates of effect sizes (e.g. Cohen's d, Pearson's r), indicating how they were calculated

Our web collection on statistics for biologists contains articles on many of the points above.

Software and code

Policy information about availability of computer code

Data collection	DigiGait and Ethovision XT software were used to acquire videos. DeepLabCut v2.3 was used to track videos. Microphotographs were analysed using ZEN Blue (Zeiss) software. SnapGene 7.2.0 software was used for cloning design.
Data analysis	Statistical analysis was performed with R Studio 2023.12.1+402 and Prism10.1.0 (264), Python script was used to calculate locomotor parameters, QuPath 0.5.1 and Fiji was used to quantify RNAscope images, motor neuron and synapse quantification was performed using Fiji 2.14.0 software. All code is available on Zenodo at this link 10.5281/zenodo.10956898, also reported in the manuscript.

For manuscripts utilizing custom algorithms or software that are central to the research but not yet described in published literature, software must be made available to editors and reviewers. We strongly encourage code deposition in a community repository (e.g. GitHub). See the Nature Portfolio guidelines for submitting code & software for further information.

Data

Policy information about availability of data

All manuscripts must include a data availability statement. This statement should provide the following information, where applicable:

- Accession codes, unique identifiers, or web links for publicly available datasets
- A description of any restrictions on data availability
- For clinical datasets or third party data, please ensure that the statement adheres to our policy

The processed data are available at PURE repository at University of St Andrews doi: https://doi.org/10.17630/940f2947-ae2b-4e18-9493-bd65612ba3a6. The videos are available under restricted access due to the dimension of the files, access can be obtained by the corresponding author. Source data are provided with

this paper.

Research involving human participants, their data, or biological material

Policy information about studies with human participants or human data. See also policy information about sex, gender (identity/presentation), and sexual orientation and race, ethnicity and racism.

Reporting on sex and gender	N/A
Reporting on race, ethnicity, or other socially relevant groupings	N/A
Population characteristics	N/A
Recruitment	N/A
Ethics oversight	N/A

Note that full information on the approval of the study protocol must also be provided in the manuscript.

Field-specific reporting

Please select the one below that is the best fit for your research. If you are not sure, read the appropriate sections before making your selection.

[x] Life sciences [ ] Behavioural & social sciences [ ] Ecological, evolutionary & environmental sciences

For a reference copy of the document with all sections, see nature.com/documents/nr-reporting-summary-flat.pdf

Life sciences study design

All studies must disclose on these points even when the disclosure is negative.

Sample size	The detailed count of males (M) and females (F) per experiment, listed for each figure, is as follows: Fig. 1d: M 14, F 20; Fig. 1g: M 11, F 16; Fig. 2k: M 0, F 8; Fig. 2x: M 6, F 6; Fig. 3e: M 9, F 9; Fig. 3n: M 9, F 11; Fig. 3o: M 5, F 4; Fig. 5u-v: M 5, F 5; Fig. 6b-e: M 19, F 16; Fig. 6f-q: M 16, F 16; Fig. 7i: M 3, F 3; Fig. 7m: M 7, F 13; Fig. 8a-m: M 6, F 10; Fig. 9a: M 4, F 6; Fig. 9b-c: M 4, F 5; Fig. 9f: M 6, F 10; Fig. 9g: M 4, F 6; S Fig. 1a: M 13, F 7; S Fig. 1b: M 14, F 0; S Fig. 1c: M 9, F 7; S Fig. 2a: M 19, F 15; S Fig. 2c-f: M 19, F 16; S Fig. 3e-t: M 3, F 1; S Fig. 4b: M 7, F 12; S Fig. 4d-i: M 7, F 13. No statistical methods were used to pre-determine sample size, but our sample size was similar to the one reported in previous publications (Allodi et al 2021 Nature Communications, Allodi et al 2016 Scientific Reports).
Data exclusions	One mouse was excluded from the study since it showed aberrant locomotor phenotype upon intraspinal injection, all other injected mice were included in the study. All experiments were performed at least in triplicates (except for the experiments were otherwise stated in the Results and Figure legend paragraphs) and observation showed reproducibility among replications. No data were excluded from the analyses.
Replication	Several trials were performed for each of the experiments (at least triplicates). For some experiments, several cohorts (independent experiments) were carried out to obtain the final data set. In all cases, attempts to replication were successful. Only PLA experiments were performed in two SOD1G93A;En1cre and two En1cre mice and 5 sections were stained per mouse.
Randomization	When applicable, mice were randomly allocated to different groups for the in vivo experiments using a block design.
Blinding	Data collection was performed blind to the condition of the experiments, although during behavioral assessment ALS mice could be recognized at later stages of disease. The analysis was automated and blind, hence the experimenter had no influence on the outcome.

Reporting for specific materials, systems and methods

We require information from authors about some types of materials, experimental systems and methods used in many studies. Here, indicate whether each material, system or method listed is relevant to your study. If you are not sure if a list item applies to your research, read the appropriate section before selecting a response.

Materials & experimental systems

n/a	Involved in the study
	x Antibodies
	x Eukaryotic cell lines
x	Palaeontology and archaeology
	x Animals and other organisms
x	Clinical data
x	Dual use research of concern
x	Plants

Methods

n/a	Involved in the study
x	ChIP-seq
x	Flow cytometry
x	MRI-based neuroimaging

Antibodies

Antibodies used	The following primary antibodies were used: DS Red (1:1000, Rabbit, #632496 Takara-Clontech), Chat (1:300, Goat, #AB144P Millipore), Vgat (1:500, Rabbit, #PA5-27569 Millipore), Esyt1 (1:100, Rabbit, #HPA0168589 Sigma), Gephyrin (1:200, Guinea Pig, #147-318-SY Synaptic Systems). The following secondary antibodies were used Alexa Fluor 488, 568, 647 concentration 1:500, Invitrogen.
Validation	All antibodies have been validated in numerous publications with IHC and Western blot on mouse samples as indicated on manifacturer's product pages and the Antibody Register.

Eukaryotic cell lines

Policy information about cell lines and Sex and Gender in Research

Cell line source(s)	293-cell line commercially available at atcc were used for co-transfection and virus generation.
Authentication	The 293-cells were not authenticated.
Mycoplasma contamination	293-cells were not tested for mycoplasma.
Commonly misidentified lines (See ICLAC register)	No commonly misidentified lines were used in this study.

Animals and other research organisms

Policy information about studies involving animals; ARRIVE guidelines recommended for reporting animal research, and Sex and Gender in Research

Laboratory animals	SOD1G93A (B6.Cg-Tg(SOD1-G93A)1Gur/J) stock no: #004435 from Jackson laboratory; wild-type C57BL6/J stock no: #000664 from Jackson Laboratories; En1cre provided by Assistant Prof. Jay Bikoff (St. Jude Children's hospital, St Louis, Texas USA). All mice were kept on a C57BL6/J background. Mice were used between postnatal day 30 and 170. Mice were housed according to standard conditions with ad libitum feeding, constant access to water, and a 12:12 hour light/dark cycle.
Wild animals	No wild animals were used.
Reporting on sex	The detailed count of males (M) and females (F) per experiment, listed for each figure, is as follows: Fig. 1d: M 14, F 20; Fig. 1g: M 11, F 16; Fig. 2k: M 0, F 8; Fig. 2x: M 6, F 6; Fig. 3e: M 9, F 9; Fig. 3n: M 9, F 11; Fig. 3o: M 5, F 4; Fig. 5u-v: M 5, F 5; Fig. 6b-e: M 19, F 16; Fig. 6f-q: M 16, F 16; Fig. 7i: M 3, F 3; Fig. 7m: M 7, F 13; Fig. 8a-m: M 6, F 10; Fig. 9a: M 4, F 6; Fig. 9b-c: M 4, F 5; Fig. 9f: M 6, F 10; Fig. 9g: M 4, F 6; S Fig. 1a: M 13, F 7; S Fig. 1b: M 14, F 0; S Fig. 1c: M 9, F 7; S Fig. 2a: M 19, F 15; S Fig. 2c-f: M 19, F 16; S Fig. 3e-t: M 3, F 1; S Fig. 4b: M 7, F 12; S Fig. 4d-i: M 7, F 13. This is also reported in the manuscript and in the Source Data File.
Field-collected samples	No field-collected samples were used.
Ethics oversight	All experiments were in accordance with the EU Directive 20110/63/EU and approved by the Danish Animal Inspectorate (Ethical permits: 2018-15-0201-01426 and 2022-15-0201-01164).

Note that full information on the approval of the study protocol must also be provided in the manuscript.

Plants

Seed stocks

Report on the source of all seed stocks or other plant material used. If applicable, state the seed stock centre and catalogue number. If plant specimens were collected from the field, describe the collection location, date and sampling procedures.

Novel plant genotypes

Describe the methods by which all novel plant genotypes were produced. This includes those generated by transgenic approaches, gene editing, chemical/radiation-based mutagenesis and hybridization. For transgenic lines, describe the transformation method, the number of independent lines analyzed and the generation upon which experiments were performed. For gene-edited lines, describe the editor used, the endogenous sequence targeted for editing, the targeting guide RNA sequence (if applicable) and how the editor was applied.

Authentication

Describe any authentication procedures for each seed stock used or novel genotype generated. Describe any experiments used to assess the effect of a mutation and, where applicable, how potential secondary effects (e.g. second site T-DNA insertions, mosiacism, off-target gene editing) were examined.
